# Hydrogen Sulfide-Releasing Indomethacin-Derivative (ATB-344) Prevents the Development of Oxidative Gastric Mucosal Injuries

**DOI:** 10.3390/antiox12081545

**Published:** 2023-08-02

**Authors:** Urszula Głowacka, Marcin Magierowski, Zbigniew Śliwowski, Jakub Cieszkowski, Małgorzata Szetela, Dagmara Wójcik-Grzybek, Anna Chmura, Tomasz Brzozowski, John L. Wallace, Katarzyna Magierowska

**Affiliations:** 1 Department of Physiology, Jagiellonian University Medical College, 16 Grzegórzecka Street, 31-531 Kraków, Poland; 2 Doctoral School of Medical and Health Sciences, Jagiellonian University Medical College, 31-530 Kraków, Poland; 3 Department of Physiology and Pharmacology, University of Calgary, Calgary, AB T2N 1N4, Canada

**Keywords:** hydrogen sulfide, non-steroidal anti-inflammatory drugs, indomethacin, ATB-344, gastric oxidative injury

## Abstract

Hydrogen sulfide (H_2_S) emerged recently as an anti-oxidative signaling molecule that contributes to gastrointestinal (GI) mucosal defense and repair. Indomethacin belongs to the class of non-steroidal anti-inflammatory drugs (NSAIDs) and is used as an effective intervention in the treatment of gout- or osteoarthritis-related inflammation. However, its clinical use is strongly limited since indomethacin inhibits gastric mucosal prostaglandin (PG) biosynthesis, predisposing to or even inducing ulcerogenesis. The H_2_S moiety was shown to decrease the GI toxicity of some NSAIDs. However, the GI safety and anti-oxidative effect of a novel H_2_S-releasing indomethacin derivative (ATB-344) remain unexplored. Thus, we aimed here to compare the impact of ATB-344 and classic indomethacin on gastric mucosal integrity and their ability to counteract the development of oxidative gastric mucosal injuries. Wistar rats were pretreated intragastrically (i.g.) with vehicle, ATB-344 (7–28 mg/kg i.g.), or indomethacin (5–20 mg/kg i.g.). Next, animals were exposed to microsurgical gastric ischemia-reperfusion (I/R). Gastric damage was assessed micro- and macroscopically. The volatile H_2_S level was assessed in the gastric mucosa using the modified methylene blue method. Serum and gastric mucosal PGE_2_ and 8-hydroxyguanozine (8-OHG) concentrations were evaluated by ELISA. Molecular alterations for gastric mucosal barrier-specific targets such as cyclooxygenase-1 (COX)-1, COX-2, heme oxygenase-1 (HMOX)-1, HMOX-2, superoxide dismutase-1 (SOD)-1, SOD-2, hypoxia inducible factor (HIF)-1α, xanthine oxidase (XDH), suppressor of cytokine signaling 3 (SOCS3), CCAAT enhancer binding protein (C/EBP), annexin A1 (ANXA1), interleukin 1 beta (IL-1β), interleukin 1 receptor type I (IL-1R1), interleukin 1 receptor type II (IL-1R2), inducible nitric oxide synthase (iNOS), tumor necrosis factor receptor 2 (TNFR2), or H_2_S-producing enzymes, cystathionine γ-lyase (CTH), cystathionine β-synthase (CBS), or 3-mercaptopyruvate sulfur transferase (MPST), were assessed at the mRNA level by real-time PCR. ATB-344 (7 mg/kg i.g.) reduced the area of gastric I/R injuries in contrast to an equimolar dose of indomethacin. ATB-344 increased gastric H_2_S production, did not affect gastric mucosal PGE_2_ content, prevented RNA oxidation, and maintained or enhanced the expression of oxidation-sensitive HMOX-1 and SOD-2 in line with decreased IL-1β and XDH. We conclude that due to the H_2_S-releasing ability, i.g., treatment with ATB-344 not only exerts dose-dependent GI safety but even enhances gastric mucosal barrier capacity to counteract acute oxidative injury development when applied at a low dose of 7 mg/kg, in contrast to classic indomethacin. ATB-344 (7 mg/kg) inhibited COX activity on a systemic level but did not affect cytoprotective PGE_2_ content in the gastric mucosa and, as a result, evoked gastroprotection against oxidative damage.

## 1. Introduction

Indomethacin (indo) is a well-known non-steroidal anti-inflammatory drug (NSAID), used as an antipyretic, anti-inflammatory, and analgesic pharmacological intervention [1]. Indo is prescribed to relieve pain and inflammation related to osteoarthritis, rheumatoid and gouty arthritis, ankylosing spondylitis, or an acutely painful shoulder [2]. However, indo is considered to have the greatest ability to cause gastric injury compared to other NSAIDs [3,4]. Indo causes gastric mucosal damage by inhibiting the activity of cyclooxygenase 1 (COX-1) that produces gastroprotective prostaglandin E_2_ (PGE_2_), decreasing bicarbonate and mucus secretion, stimulating gastric acid secretion, increasing reactive oxygen species (ROS) generation, and decreasing the level of physiological anti-oxidative molecular response [3]. NSAIDs were reported to impair gastric mucosal biosynthesis of cytoprotective hydrogen sulfide (H_2_S). H_2_S, next to nitric oxide (NO) or carbon monoxide (CO), is an endogenous gaseous mediator with anti-inflammatory, anti-oxidative, and cytoprotective properties [5,6]. H_2_S is biosynthesized mainly by three enzymes, cystathionine γ-lyase (CTH), cystathionine β-synthetase (CBS), and 3-mercaptopyruvate sulfur transferase (MPST), of which CBS and CTH are considered to be cytosolic enzymes, while MPST may be localized in both mitochondria and the cytosol [7,8]. H_2_S plays an important role in the maintenance of the integrity of the gastric mucosa [9,10]. Importantly, oxidative stress and gastric mucosal injury evoked by ischemia-reperfusion (I/R) are characterized by a sudden fall in blood supply to tissues and organs, followed by immediate restoration of blood flow and reoxygenation [11].

Under clinical conditions, I/R damage of the stomach occurs as a result of bleeding from a peptic ulcer, rupture of a vessel, surgery, ischemic disease of the GI tract, and hemorrhagic shock [12]. The mechanism of I/R damage is complex and associated with many factors, including inflammation, excessive production of ROS in the mucosa, leukocyte infiltration, and reduced NO release. However, oxidative stress seems to be predominant [13]. ROS excess causes lipid peroxidation of cell membranes, ribonucleic acid (RNA) or deoxyribonucleic acid (DNA) oxidation, and contributes to the production of toxic products such as malondialdehyde (MDA) [14,15]. On the other hand, H_2_S exhibits anti-oxidative effects due to the inhibition of ROS production, modulation of glutathione (GSH) activity, activation of the expression of antioxidant enzymes (AOE) [16,17], and enhancement of mitochondrial integrity [11]. Indeed, we reported recently that mitochondria-targeted H_2_S donor AP39 protected the gastric mucosa against gastric I/R damage [18].

To counteract the gastrointestinal (GI) toxicity of NSAIDs, H_2_S-releasing derivatives of these drugs were developed. Some of them were shown in clinical and/or preclinical studies to be GI-safe compared to the parent drugs [19,20,21]. Additionally, ATB-346 (H_2_S-releasing naproxen derivative (Otenaproxesul, Antibe Therapeutics Inc., Toronto, ON, Canada) was shown to exert chemo-preventive effects vs. colorectal cancer [22]. We reported that H_2_S-releasing ketoprofen derivative (ATB-352), unlike classic ketoprofen, is GI-safe and does not significantly affect the intestinal microbiome profile [23].

Thus, we aimed to investigate here for the first time the impact of the new hybrid NSAID, H_2_S-releasing ATB-344 vs. classic indomethacin, on gastric mucosal integrity and the capacity of gastric mucosal defense to cope with acute oxidative injury induced by I/R. We focused on the pharmacological impact of these drugs on redox balance and gastric mucosal integrity based on macro- and microscopic evaluation and the assessment of the molecular pattern of gastric mucosal barrier components.

## 2. Materials and Methods

### 2.1. Experimental Design, Chemicals and Drugs

Male Wistar rats (40) with an average weight of 220–300 g were deprived of food for 12–16 h with free access to tap water before the treatments and exposure to I/R. Regular compounds and chemicals were purchased from Sigma Aldrich (Schnelldorf, Germany) unless otherwise stated.

All procedures performed in the study were approved by the I Local Ethical Committee for Care and Use of Experimental Animals, held by the Faculty of Pharmacy, Jagiellonian University Medical College in Cracow (Decision No.: 311/2019; Date: 17 July 2019 and 661/2022; Date: 27 September 2022). The principles of the 3 Rs (Replacement, Reduction, and Refinement) were incorporated into the research design. The difference between male and female rats occurs, but it is not clearly evidenced in terms of the integrity of the gastric mucosal barrier and its resistance to NSAIDs [24]. Therefore, to reduce the number of animals, we included only male rats in this study.

Rats were randomly divided into designated experimental groups (*n* = 5 per group) and pretreated intragastrically (i.g.) using an orogastric tube with 1 mL of (1) vehicle (dimethyl sulfoxide (DMSO) and 1% sodium carboxymethyl cellulose (CMC) in water 1:9), (2) ATB-344 (7, 14, and 28 mg/kg (that equals approx. 14, 28, and 56 μmol/kg, respectively), Antibe Therapeutics, Toronto, ON, Canada), and (3) classic indo without H_2_S-releasing moiety in equimolar doses (5, 10, and 20 mg/kg (that equals approx. 14, 28, and 56 μmol/kg, respectively)).

### 2.2. I/R-Induced Gastric Lesions, Macro-and Microscopic Assessment of Gastric Damage, Tissue Collection and Storage

I/R gastric lesions were induced 30 min after the treatments, as described previously [10,25]. Briefly, under isoflurane anesthesia, the abdomen was opened, the celiac artery was clamped for 30 min (hypoxia), and then the clamp was removed (reperfusion). After 3 h of reperfusion, rats were sacrificed by i.p. administration of a lethal dose of pentobarbital (Biowet, Pulawy, Poland), and the gastric damage was measured planimetrically (mm^2^). Gastric mucosa from each rat was collected, immediately frozen in liquid nitrogen, and stored at −80 °C for further analysis. For microscopic analysis, the gastric tissue sections were excised and fixed in 10% buffered formalin, pH = 7.4. Samples were stained with haematoxylin/eosin (H&E) as described previously [26]. Digital documentation of histological slides was obtained using a light microscope (AxioVert A1, Carl Zeiss, Oberkochen, Germany) and the ZEN Pro 2.3 software (Carl Zeiss, Oberkochen, Germany) [27].

### 2.3. Assessment of H_2_S Release in Gastric Mucosa by Modified Zinc Trapping Assay and Methylene-Blue Method

H_2_S release in the gastric mucosa was determined by the modified methylene blue method, allowing for the assessment of the level of volatile sulfide release from the gastric mucosa as previously described [10,23,28,29,30,31]. Briefly, gastric mucosa was homogenized in an ice-cold 50 mM potassium phosphate buffer, pH = 8.0. Then, L-cysteine (10 mM) and pyridoxal-5′-phosphate (P5P; 2 mM) were added to the homogenate, and the vials, including inner tubes with zinc acetate (to avoid direct contact with the tissue and reaction mixture), were then incubated in a shaking water bath (37 °C) for 90 min. Next, trichloroacetic acid (TCA; 50%; 0.5 mL) was injected into the reaction mixture through a septum plug. The mixture remained to stand for 60 min at 50 °C to allow H_2_S trapping by zinc acetate. *N*,*N*-Dimethyl-p-phenylenediamine sulfate (20 mM; 50 μL) in 7.2 M HCl and FeCl_3_ (30 mM; 50 μL) in 1.2 M HCl were added to the internal tubes once separated out of the reaction mixture flask. After 20 min, absorbance at 670 nm was measured with a microplate reader (Tecan Sunrise, Mannedorf, Switzerland). The calibration curve of the absorbance as a function of H_2_S concentration was obtained using NaHS solution in various concentrations.

### 2.4. Determination of PGE_2_ Concentration in Gastric Mucosa and Serum by ELISA Test

PGE_2_ concentrations in gastric mucosa and serum were determined according to the manufacturer’s protocol (EHPGE_2_, PGE_2_ ELISA Kit, Invitrogen, Thermo Fisher Scientific, Vilnius, Lithuania) and as described in detail elsewhere [27]. Results were expressed in pg/mL of gastric tissue homogenate or serum.

### 2.5. Evaluation of 8-Hydroxyguanozine (8-OHG) Concentration in Gastric Mucosa

The content of 8-OHG in gastric mucosa as an RNA oxidative damage marker was assessed using an ELISA kit (589320, Cayman Chemical, Ann Arbor, MI, USA) and normalized to total RNA level, according to the manufacturer’s protocol and as described in detail elsewhere [18].

### 2.6. Determination of mRNA Expression for Selected Genes by Real-Time Polymerase Chain Reaction (PCR)

Total RNA was isolated from gastric mucosa using a commercially available kit with spin columns (GeneMATRIX Universal RNA Purification Kit, EURx, Gdansk, Poland) according to manufacturer protocol. RNA concentration was measured using a Nano Drop One spectrophotometer (Thermo Fisher Scientific, Waltham, MA, USA). Reverse transcription was performed using the High-Capacity cDNA Reverse Transcription Kit (MultiScribe™, Applied Biosystems, Life Technologies, Carlsbad, CA, USA). Expression of mRNA was determined using SGqPCR Master Mix (2×) with SYBR-Green (EURx, Gdansk, Poland) or 2XTaqMan Fast Advanced Master Mix (Thermo Fisher Scientific, Vilnius, Lithuania) with 20X TaqMan gene expression assays (Thermo Fisher Scientific, Vilnius, Lithuania). Expression for COX-1, COX-2, suppressor of cytokine signaling 3 (SOCS3), CCAAT enhancer binding protein (C/EBP), annexin A1 (ANX-1), hypoxia inducible factor (HIF)-1α, interleukin (IL)-1β, IL-1 receptor type I (IL-1R1), IL-1R2, tumor necrosis factor receptor 2 (TNFR2), and inducible nitric oxide synthase (iNOS) were determined using specific primers. COX-1 (Ptgs1, NM_017043.4 ) was determined using 5′-AGGTGTACCCACCTTCCGT-3′ forward and 5′-CCAGATCGTGGAGAAGAGCA-3′ reverse primers. COX-2 (Ptgs2, NM_017232.4) was determined using 5′-ATCAGAACCGCATTGCCTCT-3′ forward and 5′-GCCAGCAATCTGTCTGGTGA-3′ reverse primers. SOCS3 (XM_008768398.2) was determined using 5′-CCTCCAGCATCTTTGTCGGAAGAC-3′ forward and 5′-TACTGGTCCAGGAACTCCCGAATG-3′ reverse primers. C/EBP (NM_024125.5) was determined using 5′-TGGACAAGCTGAGCGACGAG-3′ forward and 5′-TGTGCTGCGCTCCCAGGTTG-3′ reverse primers. ANXA1 (NM_012904.2) was determined using 5′-TGAGAAGTGCCTCACAACCA-3′ forward and 5′-TCTTATGGCGAGTTCCAGCA-3′ reverse primers. HIF-1α (NM_024359.2) was determined using 5′-ATCCATTTTCAGCTCAGGACAC-3′ forward and 5′-GGTAGGTTTCTGTAACTGGGTCTG-3′ reverse primers. IL-1β (NM_031512.2) was determined using 5′-GCTATGGCAACTGTCCCTGA-3′ forward and 5′-AGTCAAGGGCTTGGAAGCAA-3′ reverse primers. IL-1R1 (NM_001412594.1) was determined using 5′-GTTTTTGGAACACCCTTCAGCC-3′ forward and 5′-ACGAAGCAGATGAACGGATAGC-3′ reverse primers. IL-1R2 (NM_001412594.1) was determined using 5′-CATTCAGACACCTCCAGCAGTTC-3′ forward and 5′-ACCCAGAGCGTATCATCCTTCAC-3′ reverse primers. TNFR2 (NM_130426.4) was determined using 5′-TGCAACAAGACTTCAGACACCGTG-3′ and 5′-AGGCATGTATGCAGATGGTTCCAG-3′ reverse primers. iNOS (NM_012611.3) was determined using 5′-TGGTGAGGGGACTGGACTTT-3′ forward and 5′-CTCCGTGGGGCTTGTAGTTG-3′ reverse primers. TaqMan Gene Expression Assays were implemented as follows: Rn07318891_s1 for CTH, Rn00560948_m1 for CBS, Rn00593744_m1 for MPST, Rn00566938_m1 for SOD-1, Rn00690588_g1 for SOD-2, Rn00567654_m1 for xanthine oxidase (XDH), Rn00561387_m1 for HMOX-1, Rn01642020_mH for HMOX-2, and Rn99999916 for GAPDH (glyceraldehyde-3-phosphate dehydrogenase), which was used as a reference gene. A PCR reaction was run using the thermal cycler Quant Studio 3 (Thermo Fisher Scientific, Waltham, MA, USA), and results were analyzed based on the ΔΔCt method where the Ct values obtained for intact gastric mucosa were used to normalize the data (except the results showed on Figure 2B where we used vehicle). A 2-fold change (with *p* < 0.05) was considered biologically and statistically significant.

### 2.7. Statistical Analysis

Results were analyzed using GraphPad Prism 9.0 software (GraphPad Software Inc., La Jolla, CA, USA). Statistical analysis was conducted using Student’s *t*-test or ANOVA with Dunnett’s multiple comparison if more than two experimental groups were compared. The Mann–Whitney test was used for the data shown on 5D. The size of each experimental group was *n* = 5, and *p* < 0.05 was considered statistically significant.

## 3. Results

### 3.1. Dose-Dependent Impact of H_2_S-Releasing ATB-344 and Indomethacin on Gastric Mucosal Integrity and H_2_S Production in Gastric Mucosa under Oxidative Stress

**Figure 1:**
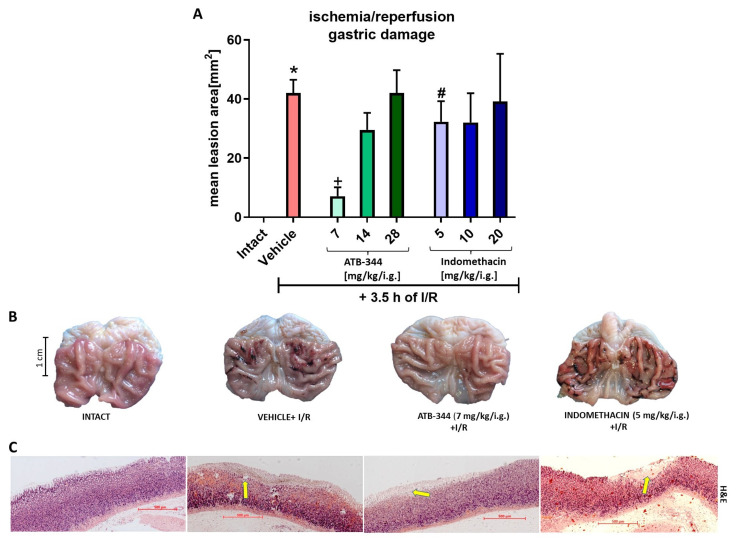
The area of gastric mucosal lesions induced by exposure to 3.5 h of I/R in rats pretreated with vehicle, ATB-344 (7, 14, and 28 mg/kg i.g.), or indomethacin (5, 10, and 20 mg/kg i.g.) (**A**). Intact refers to healthy gastric mucosa without exposure to I/R. Results are mean ± SEM of 4–5 rats per group. An asterisk (*) indicates a significant change compared to intact (*p* < 0.05). Cross (+) indicates a significant change compared to vehicle (*p* < 0.05). Hash (#) indicates a significant change between ATB-344 and indo (*p* < 0.05). Macroscopic (**B**) and microscopic (**C**) appearance of representative gastric mucosa of rats exposed or not (intact) to I/R and pretreated with vehicle, ATB-344 (7 mg/kg i.g.), or indo (5 mg/kg i.g.). Yellow arrows pointed out I/R-induced epithelial erosions. Histological slides were stained with hematoxylin and eosin (H/E).

Figure 1A shows the mean lesion area of I/R-induced gastric lesions in rats pretreated with vehicle, ATB-344 (7–28 mg/kg i.g.), or indo (5–20 mg/kg i.g.). ATB-344 applied in a dose of 7 mg/kg but not 14 and 28 significantly reduced I/R-induced gastric lesions area compared with vehicle (*p* < 0.05). Indo (5 mg/kg i.g.), significantly increased I/R-damage area compared with the equimolar dose of ATB-344 (*p* < 0.05). Therefore, ATB-344 (7 mg/kg i.g.) and indo (5 mg/kg i.g.) were further evaluated on a molecular level. Figure 1B shows the macroscopic appearance of representative gastric mucosa, exposed or not (intact) to I/R. In rats pretreated with ATB-344 (7 mg/kg) but not with vehicle or indo (5 mg/kg), gastric erosions were limited to a few hemorrhagic dot-like lesions. Figure 1C shows the microscopic appearance of gastric mucosa exposed to I/R in rats pretreated with vehicle, ATB-344 (7 mg/kg), or indo (5 mg/kg). I/R caused disruption of the mucus layer, deep epithelial damage with leukocyte infiltration, and bleeding. In ATB-344 pretreated gastric mucosa, I/R-injury was superficial without bleeding, whereas I/R-exposed gastric mucosa pretreated with indo was microscopically similar to vehicle.

**Figure 2:**
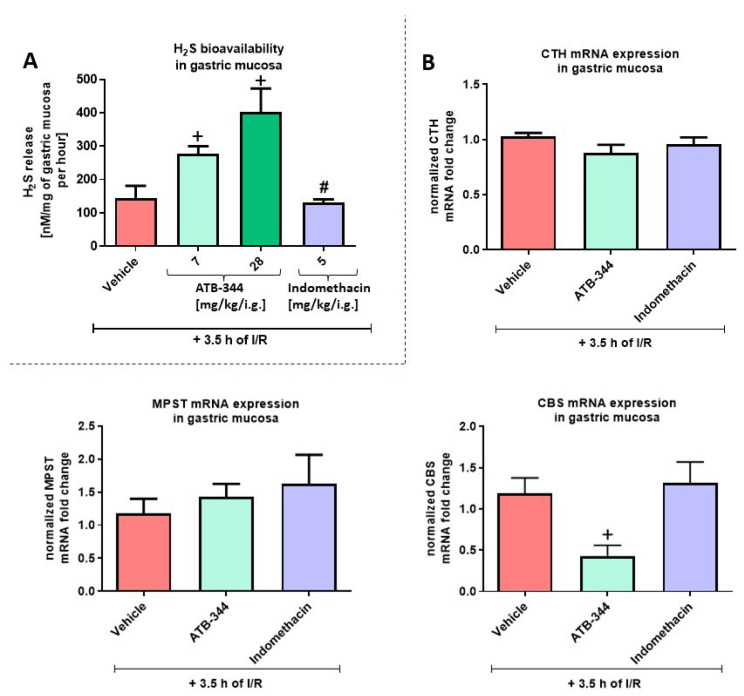
Gastric mucosal H_2_S production (**A**) and gastric mucosal mRNA expression of CTH, MPST, and CBS (**B**) in rats exposed to 3.5 h of I/R and pretreated i.g. with vehicle, ATB-344 (7 and 28 mg/kg), or indomethacin (5 mg/kg). (**A**) For gene expression analysis, ATB-344 was applied in a dose of 7 mg/kg i.g. (**B**). Results are mean ± SEM of five values per group. Cross (+) indicates a significant change compared to vehicle (*p* < 0.05). Hash (#) indicates significant changes between ATB-344 (7 mg/kg) and indomethacin (*p* < 0.05).

Figure 2A shows that the level of released volatile H_2_S was significantly increased in gastric mucosa treated with ATB-344 (7 and 28 mg/kg/i.g.) compared to vehicle (*p* < 0.05). Indo (5 mg/kg i.g.) significantly decreased H_2_S release compared with the equimolar dose of ATB-344 (*p* < 0.05) but not with vehicle. We reported previously that there is no significant difference in H_2_S release from healthy (intact) gastric mucosa vs. gastric mucosa exposed to 3.5 h of I/R [10]. Figure 2B demonstrates that ATB-344 administered in a dose of 7 mg/kg (i.g.) significantly decreased gastric mucosal mRNA expression of CBS but not CTH or MPST compared with vehicle (*p* < 0.05). We reported previously that CTH expression was elevated, while CBS and MPST expression were downregulated in gastric mucosa exposed to 3.5 h of I/R vs. healthy (intact) gastric mucosa [10].

### 3.2. Impact of H_2_S-Releasing ATB-344 and Indomethacin on Gastric Mucosal and Serum PGE_2_ Concentration and Gastric Mucosal mRNA Expression of COX-1 and COX-2

**Figure 3:**
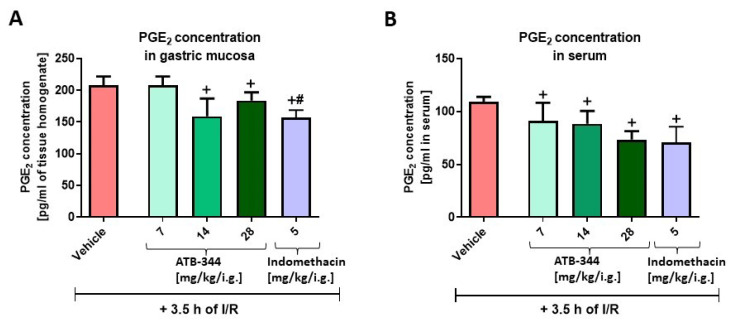
Gastric mucosal (**A**) and serum (**B**) PGE_2_ concentrations in rats exposed to 3.5 h of I/R and pretreated with vehicle, ATB-344 (7, 14, and 28 mg/kg i.g.), or indomethacin (5 mg/kg i.g.). Results are mean ± SEM of five values per group. Cross (+) indicates significant changes compared to vehicle (*p* < 0.05). Hash (#) indicates significant changes between ATB-344 (7 mg/kg) and indomethacin (*p* < 0.05).

Figure 3A shows that ATB-344 applied in doses of 14 and 28 mg/kg i.g. and indomethacin (5 mg/kg i.g.) reduced PGE_2_ concentration in gastric mucosa versus vehicle (*p* < 0.05). ATB-344 (applied in a dose of 7 mg/kg i.g.) significantly reduced PGE_2_ concentration in gastric mucosa but not in serum compared to vehicle (*p* < 0.05) (Figure 3A,B). Indo (5 mg/kg i.g.) significantly decreased gastric mucosal PGE_2_ concentration compared with an equimolar dose of ATB-344 (*p* < 0.05) (Figure 3A). We showed previously that gastric mucosal levels of PGE_2_ were decreased in gastric mucosa exposed to 3.5 h of I/R vs. healthy (intact) gastric mucosa [32]. Indo (5 mg/kg i.g.) significantly reduced serum concentrations of PGE_2_ compared with vehicle (*p* < 0.05) (Figure 3B).

**Figure 4:**
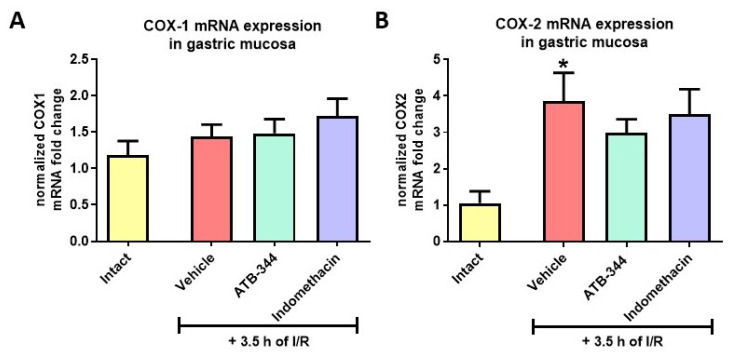
Gastric mucosal mRNA expression of COX-1 (**A**) and COX-2 (**B**) in rats exposed or not (intact) to 3.5 h of I/R and pretreated with vehicle, ATB-344 (7 mg/kg i.g.), or indo (5 mg/kg i.g.). Results are mean ± SEM of five values per group. Asterisk (*) indicates significant changes compared to intact (*p* < 0.05).

Exposure to I/R significantly elevated gastric mucosal COX-2 but not COX-1 mRNA expression vs. intact (*p* < 0.05) (Figure 4A,B). Pretreatment with ATB-344 and indo did not alter these markers compared to the vehicle.

### 3.3. Gastric Mucosal Oxidation- and Hypoxia-Sensitive Markers

**Figure 5:**
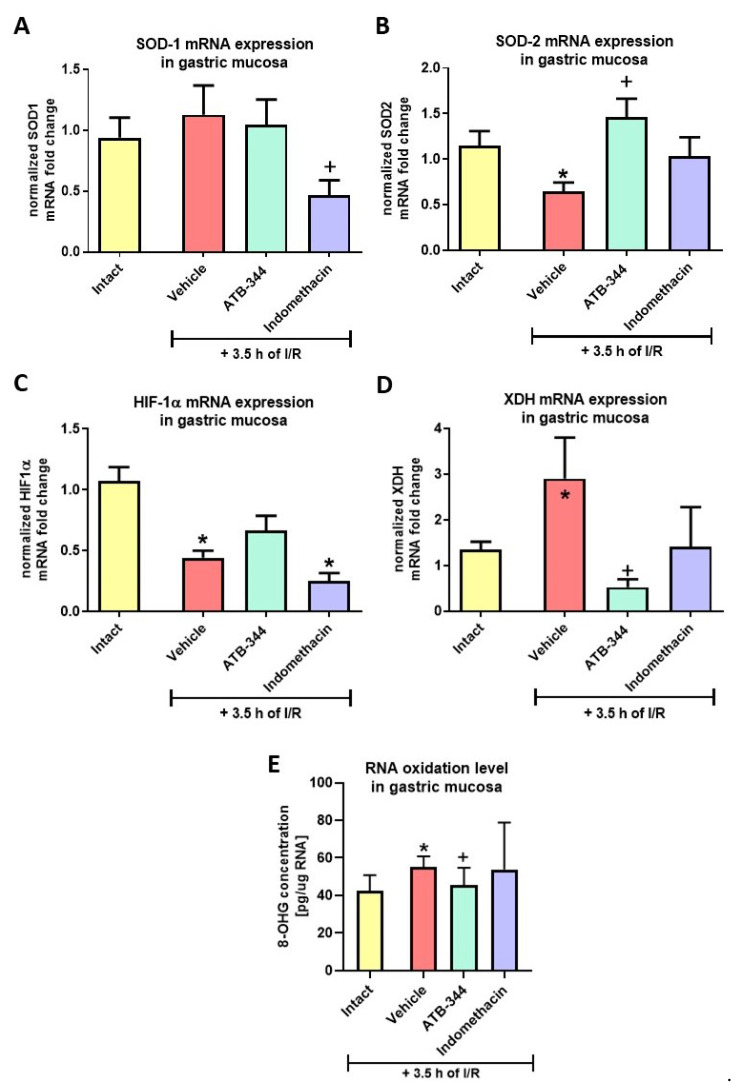
Gastric mucosal mRNA expression of SOD-1 (**A**), SOD-2 (**B**), HIF-1α (**C**), XDH (**D**), and 8-hydroxyguanozine (8-OHG) levels in gastric mucosa (**E**) of rats exposed or not (intact) to 3.5 h of I/R and pretreated with vehicle, ATB-344 (7 mg/kg i.g.), or indo (5 mg/kg i.g.). Results are mean ± SEM of five values per group. Asterisk (*) indicates significant changes compared to intact (*p* < 0.05); cross (+) indicates significant changes compared to vehicle (*p* < 0.05).

Exposure to I/R significantly decreased gastric mucosal SOD-2 and HIF-1α (Figure 5B,C) and increased XDH (Figure 5D), but did not alter SOD-1 (Figure 5A) mRNA expression vs. intact (*p* < 0.05). Respectively, pretreatment with ATB-344 increased gastric mucosal SOD-2 and reduced XDH but did not alter HIF-1α (*p* < 0.05) (Figure 5B–D). Indo decreased SOD-1 expression vs. vehicle (Figure 5A) and HIF-1α vs. intact (Figure 5C) (*p* < 0.05). Figure 5E shows that 8-OHG levels were significantly increased in gastric mucosa with I/R-induced damages compared to intact (*p* < 0.05). ATB-344 (7 mg/kg i.g.) but not indomethacin (5 mg/kg i.g.) reduced gastric mucosal 8-OHG levels compared to vehicle (*p* < 0.05).

### 3.4. Heme Oxygenase-1 as the Inducible Anti-Oxidative Marker of Gastric Mucosal Redox Imbalance and Inflammation

**Figure 6:**
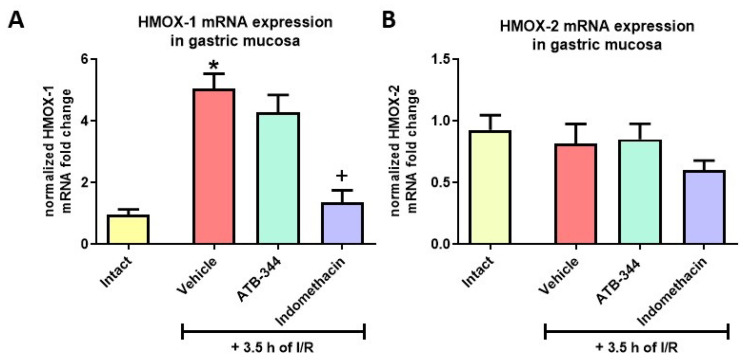
Gastric mucosal mRNA expression of HMOX-1 (**A**) and HMOX-2 (**B**) in rats exposed or not (intact) to 3.5 h of I/R and pretreated with vehicle, ATB-344 (7 mg/kg i.g.), or indo (5 mg/kg i.g.). Results are mean ± SEM of five values per group. Asterisk (*) indicates significant changes compared to intact (*p* < 0.05); cross (+) indicates significant changes compared to vehicle (*p* < 0.05).

Exposure to I/R significantly elevated gastric mucosal HMOX-1 but not constitutive HMOX-2 mRNA expression vs. intact (*p* < 0.05) (Figure 6A,B). Pretreatment with ATB-344 did not alter expression, while indo decreased HMOX-1 expression (*p* < 0.05).

### 3.5. Gastric Mucosal Markers of I/R-Related Inflammation

**Figure 7:**
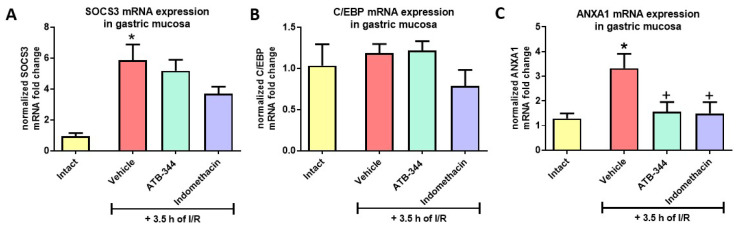
Gastric mucosal mRNA expression of SOCS3 (**A**), C/EBP (**B**), and ANXA1 (**C**) in rats exposed or not (intact) to 3.5 h of I/R and pretreated with vehicle, ATB-344 (7 mg/kg i.g.), or indo (5 mg/kg i.g.). Results are mean ± SEM of five values per group. Asterisk (*) indicates significant changes compared to intact (*p* < 0.05); cross (+) indicates significant changes compared to vehicle (*p* < 0.05).

Exposure to I/R significantly increased gastric mucosal expression of SOCS3 (Figure 7A) and ANXA1 (Figure 7C) but did not alter C/EBP mRNA expression vs. intact (*p* < 0.05) (Figure 7B). Both ATB-344 (7 mg/kg i.g.) and indo (5 mg/kg i.g.) significantly reduced the level of ANXA1 vs. vehicle (*p* < 0.05) (Figure 7C).

**Figure 8:**
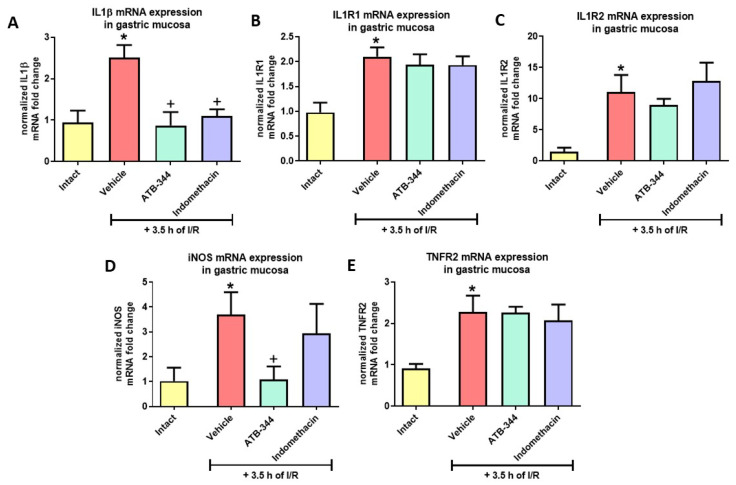
Gastric mucosal mRNA expression of IL-1β (**A**), IL-1R1 (**B**), IL-1R2 (**C**), iNOS (**D**), and TNFR2 [33,34,35,36,37] (**E**) in rats exposed or not (intact) to 3.5 h of I/R and pretreated with vehicle, ATB-344 (7 mg/kg i.g.), or indo (5 mg/kg i.g.). Results are mean ± SEM of five values per group. Asterisk (*) indicates significant changes compared to intact (*p* < 0.05); cross (+) indicates significant changes compared to vehicle (*p* < 0.05).

Exposure to I/R significantly elevated gastric mucosal expression of IL1β, IL-1R1, IL-1R2, iNOS, and TNFR2 versus intact (*p* < 0.05) (Figure 8A–E). Pretreatment with ATB-344 or indo decreased the expression of IL1β (Figure 8A), while only ATB-344 reduced iNOS vs. vehicle (*p* < 0.05) (Figure 8D).

## 4. Discussion

We demonstrated here for the first time that H_2_S-releasing ATB-344, a hybrid derivative of indo (that belongs to NSAIDs), dose-dependently enhanced gastric mucosal ability to cope with oxidative injuries [38]. We observed that, i.g. pretreatment with ATB-344 (7 mg/kg) but not an equimolar dose of classic indo, reduced the gastric damage induced by the exposure to I/R. This observation is in complete opposition to the widely observed gastrotoxicity of classic indo and other NSAID in clinical pharmacology [39,40]. On the other hand, H_2_S signaling is known to contribute to the maintenance of gastric mucosal integrity, regeneration, and oxidative balance [13,18,41]. H_2_S, as an endogenous molecule produced by the enzymatic activity of CTH, CBS, or MPST, is the main regulator of post-translational S-sulfhydration (persulfidation) of proteins that has been reported, e.g., in aging, Alzheimer’s disease, or the cardiovascular system [33,34,35,36,37,42]. Importantly, due to the development of a new methodological approach, sulfide signaling and its anti-oxidative capacity were shown to involve the generation of reactive sulfur species and persulfide or polysulfide formation, which could also be considered an H_2_S storage system [34,43,44,45,46]. We have implemented here the well-known zinc trapping assay, but with a modified protocol allowing us to assess the level of volatile sulfide released from gastric mucosa [10,28,30,31,47]. Polysulfides are not generally volatile but are a direct product of sulfide oxidation and are very unstable in a reducing environment. Therefore, we could not exclude them as possible mediators of the H_2_S-triggered activity of ATB-344 in the gastric mucosa. In fact, our data revealed that the gastroprotective dose of ATB-344 (7 mg/kg i.g.) enhanced the levels of H_2_S released in gastric mucosa (by approx. 50%) and decreased PGE_2_ content in serum but not gastric mucosa. However, the equimolar dose of indomethacin (5 mg/kg i.g.) did not elevate gastric mucosal levels of H_2_S and decreased PGE_2_ content in serum and gastric mucosa. As a result, there was no gastroprotection observed. Of note, PGE_2_ is known to contribute to the maintenance of gastric mucosal integrity, e.g., by decreasing bicarbonate and mucus secretion or by modulating gastric acid secretion [3].

We implemented here the starting dose of 5 mg/kg i.g. for indomethacin, which has been shown previously to reverse beneficial effects of possibly gastroprotective compounds when applied i.p., as a model dose in gastrointestinal pharmacology [32]. Additionally, 30 mg/kg i.g. of indomethacin is known to induce gastric mucosal damage itself, and we aimed to avoid this effect [38]. Therefore, in our study, we implemented for this NSAID a dose range of 5–20 mg/kg i.g.

Interestingly, we observed that higher doses of ATB-344 (14 and 28 mg/kg i.g.) decreased serum and gastric mucosal levels of PGE_2_. A further increase in gastric mucosal H_2_S level due to the administration of ATB-344 (28 mg/kg i.g.) did not counteract the indomethacin-triggered fall in gastric mucosal PGE_2_ content. The COX-inhibiting effect exceeded H_2_S-mediated molecular benefits and led to the loss of gastroprotective capacity at higher doses of ATB-344. Therefore, we conclude that 7 mg/kg of ATB-344 is the maximal gastroprotective dose that, due to its H_2_S-releasing properties, did not alter gastric mucosal PGE_2_ content but still maintained its ability to inhibit COX on a systemic level. At this dose, the H_2_S-releasing moiety counteracted pathogenic inhibition of COX in gastric mucosa induced by indomethacin, which evoked the gastroprotection of ATB-344 against I/R-induced gastric mucosal injury.

Our previous study revealed that the H_2_S release due to the activity of the enzymes involved in endogenous H_2_S biosynthesis (CTH, CBS, or MPST) was not affected in gastric mucosa exposed to 3.5 h of I/R [10]. At the same time, gastric mucosal expression for CTH was upregulated, while for CBS or MPST, it decreased. Elevated bioavailability of H_2_S due to, i.g. pretreatment with NaHS (as H_2_S-releasing salt) attenuated I/R-damage development [10]. In this study, we observed that ATB-344-triggered H_2_S release did not affect the expression of CTH or MPST, similarly to classic indomethacin. However, gastric mucosal expression of CBS was downregulated by ATB-344. In fact, overexpression of CBS has been suggested to contribute to the pathogenesis of various pathologies [48,49]. This is in line with the study of Scheid et al., where inhaled H_2_S prevented ischemia-reperfusion injury of neuronal tissue but also downregulated CBS expression [50]. We also previously observed the downregulation of gastrointestinal expression of CBS by the H_2_S-delivering derivative of ketoprofen (ATB-352), in parallel with elevated gastric mucosal H_2_S release, in opposition to the classic form of this NSAID [23]. Moreover, it was shown that protein expression of CTH, CBS, and MPST in gastric mucosa exposed to oxidative stress was not altered by ATB-346 (an H_2_S-releasing derivative of naproxen) that has the same H_2_S-releasing moiety as ATB-344 [51]. Taken together, we conclude that the gastroprotective effect of ATB-344 does not depend on the modulation of enzymatic H_2_S production but it is rather due to the increased level of H_2_S that is released from the appropriate chemical moiety (based on 4-hydroxythiobenzamide) of this derivative of indomethacin.

The H_2_S-releasing group combined with naproxen or ketoprofen (ATB-346 and ATB-352, respectively) was reported to enhance the GI safety of these drugs [21,23]. However, the implementation of this platform to indo remained unexplored in terms of its impact on gastric mucosal integrity under oxidative conditions. In fact, despite the very effective anti-inflammatory, anti-pyretic, or analgesic activity of NSAIDs, clinical use of these interventions is limited due to the adverse effects on the gastric mucosa, especially in individuals with aging-related disrupted GI integrity and predisposed to oxidative stress [52].

We evaluated here the pharmacological effect of ATB-344 vs. indo (applied i.g.) on gastric mucosal integrity and defense against oxidative I/R injury. We have implemented the experimental model of I/R-induced gastric damage that is based on 30 min of ischemia followed by 3 h of reperfusion. This scheme was previously shown to be optimal for testing possible therapeutic options [18]. The time point was selected based on previous studies investigating the impact of indomethacin on gastric I/R-damage and, most importantly, is supported by our recent study on the impact of NaHS on the course of I/R-gastric mucosal damage in a time-dependent manner [10,38]. Decreased blood supply to the gastric tissues causes cell dysfunction and, during prolonged ischemia, leads to cell death, e.g., as a result of bleeding from a peptic ulcer or hemorrhagic shock [53]. Paradoxically, after reperfusion, pre-existing damage deepens. Excessive production of ROS is considered a critical factor in the development of reperfusion injury [54]. In ischemic tissues, accumulation of adenosine and hypoxanthine—a substrate for xanthine oxidase (XDH) is well recognized as the major source of cellular ROS predominantly raised by reperfusion [54]. Indeed, during reperfusion, hypoxanthine is metabolized to xanthine, forming ROS [55]. In animal studies of I/R injury, allopurinol (XDH inhibitor) has been shown to reduce the damage, improve functional response after I/R injury, and decrease the scale of oxidative stress [56,57].

We observed in this study that ATB-344-mediated gastroprotection was accompanied by changes in crucial molecular targets levels reflecting the status of gastric mucosal integrity. We showed that H_2_S-releasing ATB-344 (7 mg/kg i.g.) but not indo (5 mg/kg i.g.) inhibited I/R-induced upregulation of gastric mucosal XDH expression and downregulation of antioxidative SOD-2. SOD activity is a key protective cellular response against ROS [58,59]. SOD-2 is the mitochondrial isoform of this antioxidative enzyme that efficiently converts superoxide to less reactive hydrogen peroxide (H_2_O_2_) and scavenges superoxide radicals [60,61]. A deficiency of SOD-2 in the mitochondria may increase the production of ROS and interfere with mitochondrial metabolism and cellular redox balance [62].

The cellular response to hypoxia involves alterations in the expression profiles of various genes, including HIF [63]. The stability and activity of HIF-1α are regulated by a plethora of post-translational modifications, including hydroxylation, acetylation, and phosphorylation [64]. Numerous animal and in vitro studies indicated that the activation of the HIF axis might protect against I/R damage, but this effect is time-dependent [41,42]. It is suggested that controllable enhancement of HIF-1α expression could be used as a therapeutic strategy to treat or prevent ischemic damage [65]. In our study, we confirmed previously observed downregulation of HIF-1α expression in gastric mucosa exposed to I/R. Indo, in contrast to ATB-344, enhanced this decline. Finally, our data revealed that ATB-344 (7 mg/kg i.g.) in contrast to indomethacin (5 mg/kg i.g.) decreased gastric mucosal RNA oxidation induced by exposure to ischemia/reperfusion. This confirms the antioxidative properties of ATB-344. Therefore, we conclude that H_2_S released from ATB-344 evoked gastroprotection followed by the enhanced defensive capacity of the gastric mucosa that prevented I/R-induced hypoxic and oxidative alterations reflected by the expression of SOD-1, SOD-2, XDH, and HIF-1α and decreased levels of RNA oxidation.

Gastric mucosal I/R injury triggers an inflammatory response expressed by the expression of inflammatory genes such as, e.g., iNOS, COX-2, and IL-1. Additionally, COX inhibition is the pharmacological target for indo and other NSAIDs [66]. Gemici et al. have found that gastric I/R increased neutrophil infiltration and iNOS protein expression [67]. Next to ROS, reactive nitrogen species (RNS) are also involved in the development of gastric I/R [68]. Moreover, NO can react with ROS to form toxic substances such as peroxynitrite and singlet oxygen [68,69]. Oxidative stress itself upregulates COX-2 and iNOS expression [55,70]. Arachidonic acid is a substrate for inflammation sensitive prostaglandins via the enzymatic activity of COX and free oxygen radicals [55,70,71]. In this study, we showed that gastric I/R increased the gastric mucosal expression of COX-2, IL-1β, IL-1R1, IL-1R2, TNFR2, and iNOS. Both ATB-344 and indo reduced the expression of inflammation-sensitive markers, but only ATB-344 decreased iNOS mRNA fold change in parallel with its gastroprotective effect. Indeed, iNOS inhibitors are considered useful agents to ameliorate the damage and dysfunction of various organs caused by I/R [71,72]. Interestingly, I/R injury activated the upregulation of anti-inflammatory SOCS3 and ANXA1 in a pathology-counteracting manner. H_2_S-releasing ATB-344, but not indo, maintained elevated expression of SOCS3. We assume that anti-inflammatory activity for both compounds was similar, but ATB-344 additionally reduced the expression of iNOS as a possible source of RNS and enhanced anti-inflammatory SOCS3.

Heat shock proteins (HSPs), such as HMOX-1, are molecular chaperones produced in response to oxidative stress, including I/R [73,74]. HMOX-1 is considered a cytoprotective pathway that is activated by harmful factors, such as I/R, and plays a protective role in the cellular defensive response to ROS-induced injury [75]. Importantly, H_2_S gastroprotection was shown to be dependent on CO bioavailability [76]. Our previously published data revealed that the GI safety of ATB-346 (an H_2_S-releasing naproxen derivative) or ATB-352 (an H_2_S-releasing ketoprofen derivative) was accompanied by enhanced mRNA and/or protein expression of HMOX-1 [23,51]. We reported here that, in contrast to classic indo, H_2_S-releasing ATB-344 maintained I/R-induced overexpression of HMOX-1 that was accompanied by decreased gastric I/R damage. We are aware that our observation is limited to the evaluation of gastric mucosal mRNA expression of HMOX-1/2. However, based on this and previously published data, we conclude that HMOX-1 activity could be the crucial mechanistic target determining the beneficial effects or GI safety of H_2_S-releasing NSAIDs.

**Figure 9:**
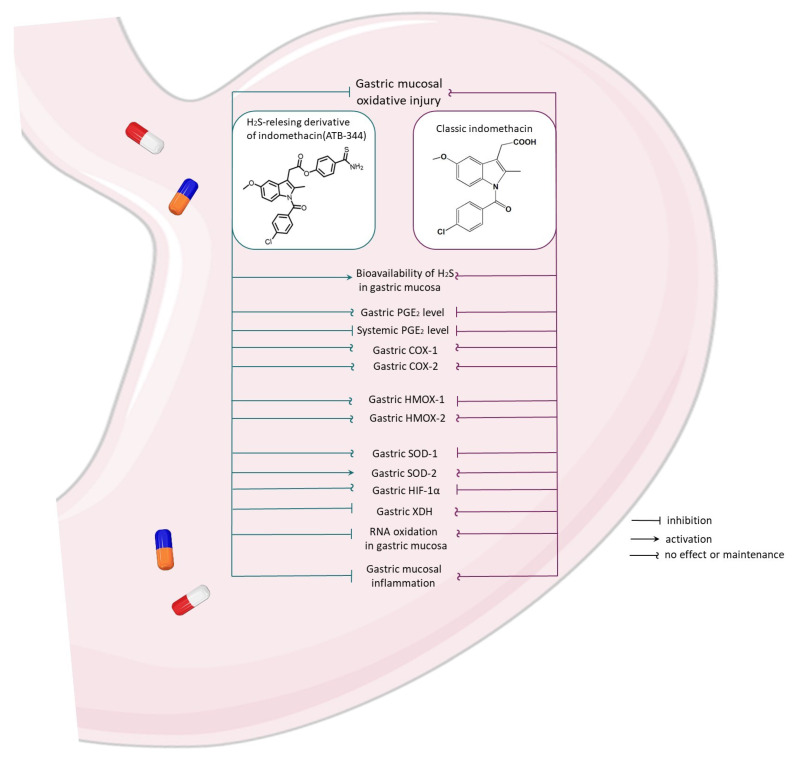
Schematic comparative overview of the main molecular effects of H_2_S-releasing ATB-344 and classic indomethacin during the development of oxidative gastric mucosal injuries.

In summary, we showed that H_2_S-releasing ability evoked the beneficial effects and GI safety of ATB-344. Precisely, ATB-344 applied i.g. in a low dose of 7 mg/kg, enhanced gastric mucosal defense against oxidative injury induced by exposure to gastric I/R. This effect was not observed for higher doses of ATB-344 (14 and 28 mg/kg) or for all equimolar doses of classic indo (5, 10, and 20 mg/kg). We assume that the effects of ATB-344 were due to H_2_S delivery rather than modulation of endogenous H_2_S production. H_2_S-releasing moiety counteracted pathogenic inhibition of COX activity and the fall in cytoprotective PGE_2_ generation in gastric mucosa induced by classic indomethacin and higher doses of ATB-344. This phenomenon evoked the dose-dependent gastroprotection of ATB-344 against I/R-induced gastric mucosal injury and, importantly, maintained its capacity to inhibit COX at the systemic level. We also conclude that the predominant anti-inflammatory and anti-oxidative capacity of ATB-344 to cope with oxidative GI lesions and gastric mucosal RNA oxidation could involve the maintenance of HMOX-1 and mitochondrial SOD-2 mRNA expression. These effects were summarized on the Figure 9. Taken together, we confirmed that H_2_S-releasing moieties conjugated with NSAIDs or other drugs are still promising targets for GI pharmacology and anti-oxidative therapeutic alternatives development.

## Data Availability

Data is contained within the article.
